# Qualitative interview study exploring Maltese veterinarians’ practice of behavioural medicine

**DOI:** 10.1002/vetr.5497

**Published:** 2025-06-06

**Authors:** Maria Debono, Amy Miele, Belinda Vigors

**Affiliations:** ^1^ Royal (Dick) School of Veterinary Studies University of Edinburgh Easter Bush UK; ^2^ Independent researcher Ellesmere UK

## Abstract

**Background:**

Veterinary behavioural medicine (VBM) is an emerging discipline, and behavioural support offered by veterinarians has been shown to positively impact One Welfare. However, the practice of VBM by veterinarians is reportedly limited. Research in this field is scant and predominantly quantitative in nature. This qualitative study aimed to explore the attitudes of small animal veterinarians in Malta towards VBM and canine mental health, their practice of VBM and any related barriers.

**Methods:**

Qualitative semi‐structured interviews were conducted, and a template analysis approach was applied to the collected data.

**Results:**

Eleven Malta‐based veterinarians were interviewed. Their responses suggest a positive regard towards canine mental health, with opportunities for improvement in VBM practice. Potential barriers to the practice of VBM included knowledge gaps, time constraints, difficulties in communicating with clients, limited availability of trusted behavioural service providers (BSPs), challenges in establishing effective working relationships with BSPs and lack of BSP regulation.

**Limitations:**

The generalisability of the findings is limited due to the nature of the study, and recruitment bias was possible.

**Conclusion:**

This study highlights avenues for changes in veterinary practices that could positively impact One Welfare. Improving veterinary knowledge of VBM and regulating BSPs are suggested as key factors in achieving this goal.

## INTRODUCTION

Veterinary behavioural medicine (VBM) is an emerging branch of veterinary medicine that is concerned with the diagnosis, treatment and prevention of behaviour problems in animals.^1^, ^2^ Behaviour problems in animals can indicate an underlying physical or mental health issue.^3^ Since animal mental health and behaviour, alongside physical health, are important determinants of animal welfare,^4^ the practice of VBM by veterinarians is becoming increasingly relevant^5^ as it can facilitate a humane approach to veterinary practice.^2^, ^6^ While pursuing further education in VBM in order to provide time‐intensive therapeutic behavioural consults may be difficult for veterinarians in primary practice, it has been suggested that the adoption of behavioural wellness concepts is achievable and essential.^7^ This may involve routine behavioural enquiry, behaviourally aware approaches to consults, provision of behavioural preventive and first‐aid advice, and timely referral to appropriate behaviour professionals.^7^ This approach can positively impact animal and human welfare, as described by the One Welfare framework.^6^

The importance of the inclusion of VBM in veterinary practice is further emphasised by client demand for such services,^8^ which is expected to increase due to the globally rising pet population,^9^ the impact of COVID‐19 restrictions on puppy behavioural development^10^ and the increasing moral concern for dogs.^11^, ^12^

Given the limited practice of VBM reported across studies to date^2^, ^13^, ^14^, ^15^, ^16^, ^17^, ^18^ and the various methodological limitations of such studies, further exploration of this area is warranted. Specifically, most studies adopted a largely quantitative, closed‐question survey approach,^2^, ^13^, ^14^, ^15^, ^17^, ^18^ potentially limiting discovery of alternative responses.^19^ Through the use of open questions and probing follow‐up questions based on participant responses, a qualitative interview approach can result in richer data, discovery of novel views and an improved understanding of the situation under study.^20^

Furthermore, geographical variation in veterinary knowledge and experience of VBM^1^ precludes generalisability of studies to date, since these have focused on veterinary populations in the UK, Ireland and the United States. Research in the veterinary and animal welfare field, including research exploring the practice of VBM, is particularly lacking in Malta, an archipelago located in the Mediterranean Sea. Malta has been facing distinct challenges in relation to animal welfare. Specifically, there is increasing public concern towards animal welfare, with the need for animal welfare education and research being recently highlighted by the government.^21^ Furthermore, Malta is facing a dog shelter overpopulation problem.^22^, ^23^ Since canine behavioural issues have been recognised as a significant cause of relinquishment in various other countries,^24^, ^25^, ^26^ exploring the practice of VBM among Malta‐based veterinarians could potentially highlight areas requiring improvement that could in turn impact the canine shelter population. Additionally, the increasingly urbanised Maltese environment^27^ may facilitate the development of various canine behavioural problems.^28^, ^29^ For instance, it can limit opportunities for daily exercise for people^27^ and, arguably, for their dogs, potentially predisposing dogs to anxiety and noise sensitivity.^30^ This, alongside the shortage of veterinary behaviour specialists in Malta, may place additional pressures on general practice veterinarians that are worthy of exploration.

The importance of veterinary practice of VBM, existing study limitations and the generally scant research in this field were therefore the driving factors behind this qualitative study, which aimed to gain a deep understanding of the attitudes of small animal veterinarians in Malta towards VBM and canine mental health, their practice of VBM and any related barriers.

## METHODS

The research team consisted of a Master's student (primary researcher and interviewer) supervised by two academics with expertise in VBM and qualitative methodology, respectively.

A phenomenological approach informed by subtle realism was adopted. While phenomenology seeks to explore the lived experiences of individuals,^31^ subtle realism acknowledges that while reality exists independently, our knowledge of it is always mediated through interpretation.^32^ Thus, rather than striving for objective truth, this study aimed to understand how veterinarians in Malta perceive, interpret and implement VBM in their daily practice, as well as the challenges they encounter. By focusing on lived experience, this study sought to shed light not only on individual perspectives but also broader structural and cultural factors influencing participants' veterinary practice in relation to VBM.

Eleven small animal veterinarians practising in Malta were interviewed using a semi‐structured qualitative approach between January and March 2022.

This study was prepared in adherence to the COREQ checklist.^33^

### Recruitment

The Malta Veterinary Surgeons’ Council and Malta Veterinary Association were engaged to disseminate an email invitation to all Malta‐registered small animal veterinarians. This invitation contained a link to an online survey. The survey informed potential participants about the study and requested interested respondents to provide demographic data, including sex, age and date of obtaining their veterinary qualification. Prospective participants were also informed of the interviewer's identity to mitigate potential discomfort during interviews, since the interviewer was a Maltese veterinarian.

Twenty veterinarians submitted a survey response. Of these, 18 participants expressed an interest in participating in the study as interviewees, and 11 (five females and six males) were selected for interview using maximum variation sampling. This involves selection of participants with varied demographic data to enable the collection of more varied insights.^34^ Since regard for animal welfare and VBM varies by sex and age,^13^, ^35^ these two demographic characteristics were used to select participants. In addition, veterinary qualification date was used to recruit participants who obtained their veterinary undergraduate degree in varied date ranges. This demographic was used as the move towards the inclusion of VBM in veterinary curricula is relatively recent^1^; thus, exposure during the undergraduate veterinary programme may vary based on time since qualification, potentially impacting participants' attitudes. Disaggregated participant demographics can be viewed in Table 1.

**TABLE 1:** Participant demographics.

Participant number	Sex	Age group (years)	Date of obtaining veterinary qualification (range)
1	F	30‒39	2001‒2010
2	F	30‒39	2011‒2020
4	M	40‒49	2001‒2010
6	M	30‒39	2011‒2020
7	F	30‒39	2001‒2010
8	F	21‒29	2011‒2020
9	M	40‒49	1991‒2000
10	M	40‒49	1991‒2000
11	M	60+	1981‒1990
13	F	50‒59	1991‒2000
20	M	30‒39	2011‒2020

### Interviews

Online semi‐structured in‐depth interviews took place using the Blackboard Collaborate platform.^36^ Only the participant and primary researcher were present during the interview. Permission to audio‐visually record the interview was requested before the interview commenced, and the participant was notified when the recording started and stopped. On average, interviews lasted 66 minutes, ranging from 54 minutes to 1 hour 30 minutes in duration.

Interviews were conducted using an interview guide consisting of several open‐ended questions and follow‐up probing questions (Table 2). The interview guide was prepared in the English language, using Kallio et al.’s framework for interview guide development,^37^ as outlined in Appendix 1. The interview guide was pilot tested with two non‐veterinarians for clarity. Amendments in wording and choice of questions followed. The guide was then pilot tested with two non‐participating small animal veterinarians practising outside of Malta.

**TABLE 2:** Indicative interview guide.

Key interview themes	1. Veterinarians’ attitudes towards canine mental health and the field of veterinary behaviour medicine	2. Awareness and knowledge of veterinarians’ role in behaviour problem prevention and early intervention Practise of behavioural medicine Relative interest in the behaviour field	3. Barriers to behavioural service provision
Interview question and probe examples	Can you tell me what the term ‘canine wellbeing’ means to you? Probe: Can you please describe some of the things that you feel are essential to canine wellbeing.	Can you describe your approach to a first puppy visit? Probe: Can you tell me what general advice you would give to the owner during their first visit?	Can you recall a time when a dog owner visited for a behavioural issue? Probe: Can you tell me more about it?

These pilot interviews were transcribed and analysed, and amendments to the guide were made accordingly. The English language interview guide was then translated into Maltese to offer interviewees the option to conduct the interview in the English or Maltese language. The Maltese language interview guide was face validated by an English and Maltese‐speaking lawyer experienced in translation. It was also tested with two non‐veterinarian native Maltese speakers for clarity. One interview was conducted in Maltese while the rest were conducted in English.

Semi‐structured interviews, guided by open‐ended questions, aimed to elicit rich, first‐person narratives. The use of a guide to conduct semi‐structured interviews promoted consistency in key areas to be covered between participants, while allowing the necessary flexibility for participants to discuss additional relevant topics and for the researcher to be responsive to participants, by for example, changing the order in which questions were posed depending on participant narrative. The semi‐structured interviewing approach using open‐ended questions also had the advantage of allowing participants the freedom to express themselves, while reducing the likelihood of incidental disclosure of sensitive information that could result in participant/interviewer discomfort and animal welfare or fitness to practice issues. The full interview guide can be found in Appendix 2.

Interviews were conducted until the interviewer was confident that data saturation had been reached. This was regarded as ‘the point at which no further significant variability emerged as a result of conducting further interviews’.^38^ The interviews were transcribed, with the one in Maltese being translated by the researcher to English, and coded through template analysis.^32^, ^39^

**FIGURE 1:**
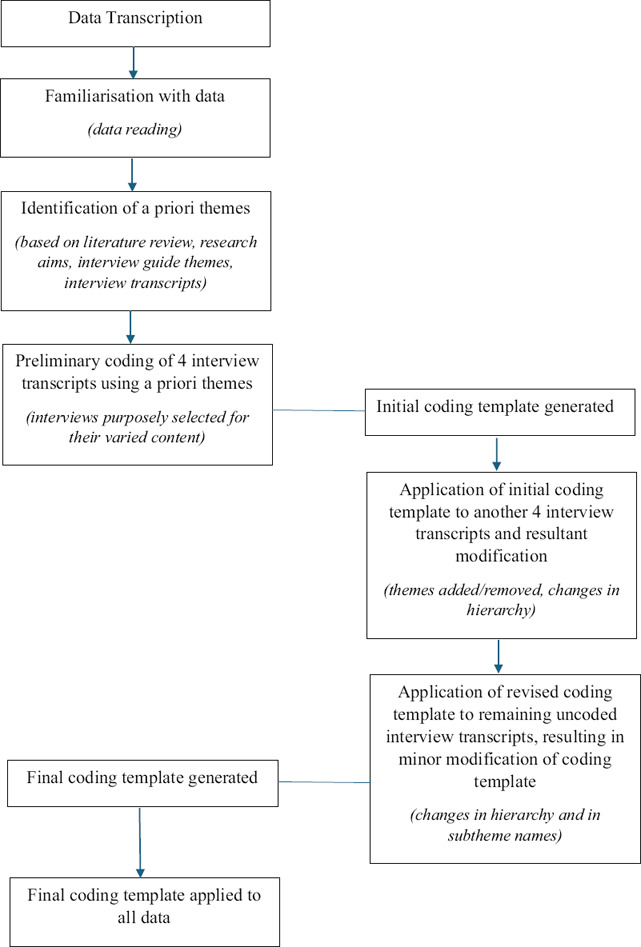
Template analysis based on the guidelines by King^39^ and Brooks et al.^32^

Template analysis ‘is a style of thematic analysis’ involving the creation of a coding template based on a sub‐set of the data and modified accordingly, before being applied to the rest of the data.^39^ First, a priori themes were identified, based on literature review, research aims and interview guide themes. A priori themes were used to ensure the inclusion of issues that are deemed to be important in relation to the topic under study, and also to facilitate preliminary coding. All interview transcripts were read twice to aid data familiarisation. Preliminary coding of four interview transcripts was conducted on NVivo software. These interviews were purposely selected for their varied content, allowing for a more comprehensive initial coding template to be developed.^32^ The coding template was applied to another four interviews and modified as needed through the inclusion of additional themes, removal of redundant themes and changes in hierarchy. The same procedure was carried out when applying the revised coding template to the remaining uncoded transcripts (Figure 1).

No further overarching themes arose following the coding of five interviews and required coding template changes past this stage involved adjustments in hierarchy and sub‐theme names. This suggested that data saturation had been reached during the interview phase in terms of major themes. However, recognising that the use of a priori themes and an initial coding template could potentially limit a truly phenomenological approach, researcher reflexivity was employed to remain open to additional insights and refinements in the coding structure, ensuring the coding template did not become prematurely fixed in the data analysis process.^32^ The final coding template can be viewed in Appendix 3.

### Reflexivity

The researcher is an integral part of the qualitative research process and thus personal biases can influence the research at any stage.^40^, ^41^ Reflexivity was particularly important due to the interviewer's background as a veterinarian from Malta with postgraduate education in VBM, which could have influenced both the data collection and analysis in several ways. For example, the interviewer's expertise in VBM could have affected verbal and non‐verbal communication during interviews and the interview narrative. A reflexive approach was thus planned and implemented throughout the research project, as suggested by Roberts^41^ and Whiting.^42^ This included the adoption of a team‐based approach involving discussion and reflection on the interview questions to be included and their wording. The use of a journal throughout the study facilitated self‐reflection and ongoing awareness of perceived personal biases that could influence interview conduction and analysis.

## RESULTS

The findings of this study revealed positive attitudes towards canine mental health but a limited practice and awareness of VBM. Table 3 presents the main themes. The following section will present and explore each theme in further detail by drawing on the perceptions of participants.

**TABLE 3:** Main themes and sub‐themes.

Theme	Type of theme (a priori or emerging)
Attitudes towards canine mental health Canine mental health is important for canine wellbeing	A priori theme Emerging sub‐theme
Practice of veterinary behavioural medicine Approach to canine physical examination Provision of behavioural advice Awareness of relevance of VBM in general veterinary practice	A priori theme Emerging sub‐theme Emerging sub‐theme Emerging sub‐theme
Attitudes towards field of veterinary behavioural medicine I am not a behaviourist	A priori theme Emerging sub‐theme
Barriers to the practice of veterinary behavioural medicine Limited veterinary enquiry and behavioural advice provision Behaviour referral options (reasons for referral or non‐referral, attitudes and emotions surrounding referral, lack of behaviour referral options/lack of clarity about available options impacting veterinarians’ wellbeing—feeling stuck and unsupported) Veterinarian‒client communication Beliefs Knowledge of VBM Lack of time	A priori theme Emerging sub‐theme Emerging sub‐theme Emerging sub‐theme Emerging sub‐theme Emerging sub‐theme Emerging sub‐theme

Abbreviation: VBM, veterinary behavioural medicine.

### Attitudes towards canine mental health

All participants regarded canine mental health as an important contributor to canine wellbeing. While some made direct reference to the term ‘canine mental health’, others illustrated its importance in the context of freedom from negative emotional and physical states:

‘… a life free of pain … hunger … from any situation that makes a dog uncomfortable and scared or anxious’. (P1)

Or by considering positive emotional and physical states:

‘… an environment which provides … interaction with humans … and maybe other animals … Care, affection, love …’ (P10)

### Practice of veterinary behavioural medicine

While an overall concern with providing low‐stress veterinary visits emerged, integration of aspects of VBM to assist with achieving this was generally limited. For example, a degree of awareness of the principles of low‐stress handling was evident. Where physical restraint was challenging, sedation or foregoing the physical examination was commonly considered a more welfare‐friendly alternative to excessive restraint. In first puppy visits, using treats, play and limiting pain was viewed as important in safeguarding patient welfare and facilitating future encounters.

Nevertheless, a degree of physical restraint was perceived to be acceptable and inevitable during physical examinations. Just three participants reported using treats to distract adult dogs during the examination. Long‐term plans to minimise future need of physical restraint, such as cooperative care training or desensitisation/counterconditioning to the veterinary clinic, were not mentioned by respondents, except for a brief indirect reference by one participant:

‘… bring it here … even for a weight check, so that he gets used to the place’. (P6)

Additionally, the practice of VBM appeared limited in terms of preventive behavioural advice provision. Advice reportedly offered during a first puppy visit generally consisted of structured preventive physical health advice, followed by some behaviour‐related advice. Participants’ behaviour‐related advice reportedly involved stating the importance of some of the following: obedience training using positive reinforcement methods, toilet training, socialisation, daily separation training, crate training and provision of environmental enrichment. Behaviour guidance reportedly provided to clients was generally not detailed:

‘… I don't go into great detail on animal behaviour. If I notice a problem, I tell them … contact an animal behaviourist’. (P6)

Where puppy socialisation was mentioned, participants generally focused on stating its importance with clients, and ways of how adequate socialisation could be achieved were seldom reported to be discussed.

Opportunities for improving awareness of participants’ potential role in the prevention of behavioural problems thus emerged. This was also apparent when participants discussed canine relinquishment. Despite aggressive behaviour being commonly considered as a main reason for relinquishment, the potential role of veterinarians in the prevention of such behavioural problems was not mentioned.

### Attitudes towards the field of veterinary behavioural medicine

Participants were not familiar with the term ‘veterinary behavioural medicine’. When referring to this field, the terms ‘animal behaviour’ or ‘behaviour’ were mainly used. The terms ‘trainer’ and ‘behaviourist’ were frequently used interchangeably, and the role of a behaviourist was often referred to as principally involving dog training, for example:

‘I studied to be more of a clinician, not to be an animal behaviourist, not to stay talking about how you should train them’. (P6)

A divide between the ‘clinical’ and the ‘behavioural’ also emerged. Participants appeared to frame VBM as a distinct field that was external to veterinary medicine:

‘I did not become a veterinarian to get into this type of work. It is not my area, I don't feel I should be the one giving this advice … I mean it is another field, another discipline in itself’. (P6)

This distinction was also evident in participant descriptions of the role of a veterinarian and that of a behaviourist, with the statement ‘I am not a behaviourist’ being used by most participants when justifying their limited practice of VBM:

‘… I mean, I do recognise my limits. As I said before, I am not a behaviourist’. (P1)

### Barriers to the practice of VBM

#### Limited veterinary enquiry and behavioural advice provision

Provision of structured behavioural first‐aid advice appeared to be lacking, with immediate referral to a behaviourist being reported as the primary recommendation in cases involving aggressive behaviour. For example:

‘I didn't give him (caregiver of dog showing aggressive behaviour at home) any advice then, for sure not’. (P9)

‘… I tell them it is better to … contact an animal behaviourist, if there is an aggression problem’. (P6)

Where further advice was given, this was described as minimal and was reportedly provided following limited enquiry.

#### Behaviour referral options

The lack of veterinary behaviourists and the unregulated status of behavioural service providers (BSPs) in Malta emerged as significant challenges in participants' approaches to behaviour cases.

Directing behavioural cases to BSPs was mentioned as an option by nine respondents, especially in cases of aggressive behaviour, with the other two respondents being reluctant to direct to BSPs. The procedure of directing a client to a BSP involved the veterinarian advising caregivers to contact an unspecified or a named non‐veterinary behaviourist. Participants’ reasons for not directing clients to a particular BSP, or not directing clients at all, included an inability to determine competence and reluctance to assume responsibility for the choice of BSP:

‘Because referring carries with it a certain amount of responsibility, you know, so I'd rather not refer, than refer to the wrong person’. (P10)

Participants generally did not make direct contact with BSPs at any point, with most relying on client updates to learn about case progress. One participant reflected on this approach:

‘To be perfectly honest with you, it's not truly a referral … I give them the number, and they contact directly … And most likely, they'll just forget about it and not do it at all’. (P8)

Limited trust in available BSPs and a general feeling of dissatisfaction with the behaviour service provided emerged. Reported reasons for this included scant positive client feedback, suspected prescription of medication by some non‐veterinary BSPs, unclear BSP options, questionable competence and lack of regulation:

‘At the end of the day, there is no regulation. There's no body’. (P2)

The lack of regulation of BSPs in Malta, together with the generally limited communication between BSPs and participants, led some participants to express how unsupported they felt when facing behavioural issues:

‘But then again … when do I refer and to whom do I refer behavioural issues? So you tend to do the bit you know with … a lot of reluctance and a lot of question marks going on in my head’. (P2)

#### Veterinarian–client communication barriers

A fear of clients interpreting behaviour‐related advice as criticism was described by some respondents as a reason behind their curtailed behavioural advice. Also, some participants appeared to express negative moral judgement towards owners of dogs showing aggressive behaviour, which may have impacted veterinarian‒client communication, for example:

‘And I really like gave her a bit of a telling off’. (P13)

‘… you are ruining this dog and he will be unadoptable because of you …’ (P8)

#### Beliefs as barriers

The belief that first‐time owners required more information than experienced dog owners was expressed by several respondents:

‘If it's a client and it's the first time … they have a dog … I definitely go through things in a lot more detail’. (P2)

Another belief held by several participants was that aggressive behaviour in puppyhood signified the presence of a hereditary aggressive trait, which appeared to lead to acceptance of aggressive behaviour:

‘I can tell you that the aggressiveness started immediately … And honestly, we joke about it with the owners because, I mean, the reaction is expected’. (P10)

#### Knowledge limitations

Knowledge limitations of VBM were evident in the approach to daily practice and to behavioural cases. Furthermore, a belief in the necessity of establishing dominance in the human–dog relationship emerged in the accounts of male participants, for example:

‘I told her that she must show dominance. That is … no fear of the dog’. (P9)

Limited familiarity with the adequate use of behaviour‐modifying drugs also emerged and appeared to deter several participants from prescribing these.

Feelings of self‐doubt in relation to VBM knowledge were expressed, suggesting knowledge limitations:

‘… am I saying the right things? … is this the right advice …?’ (P2)

Knowledge limitations were also frequently self‐declared:

‘One of the biggest … limitations I have is that I feel I don't know enough sometimes myself to give enough advice’. (P4)

Knowledge limitations appeared to negatively impact participants’ comfort level when dealing with behaviour issues: out of 11 participants, eight stated that they felt less comfortable discussing behavioural issues when compared to physical health issues. Many participants felt that the minimal exposure to VBM in their veterinary undergraduate degree significantly contributed to their limited knowledge of VBM.

#### Time‒knowledge interplay as a barrier

Many respondents described how feeling time constrained affected their approach, for example:

‘… considering the limited time of a medical examination … the approach is … a bit more imposing …’ (P10)

Viewing VBM practice as an in‐house service, with limited clarity on its potential integration into general veterinary practice, may further explain why time was often considered as a barrier, as reflected below:

‘A behavioural consult takes a long time. You can't do it in 15 minutes’. (P7)

‘It (VBM) involves a lot of communication with the owner. I don't have the time for that’. (P10)

Additionally, though a self‐declared interest in ‘behaviour’ as a field emerged among participants, this did not often translate into significant study of VBM. Feeling generally time constrained appeared to instigate the prioritisation of study in other areas of veterinary medicine. Also, the view of the practice of VBM as time consuming appeared to deter pursuit of further education in VBM:

‘And so, automatically, even though as a subject, it does interest me, knowing that … I don't have the time to give the best I can … I try to use my time reading about … other fields in veterinary medicine’. (P10)

## DISCUSSION

Despite a positive attitude towards canine mental health, the practice of VBM among this study population appears to be limited. This is consistent with the findings of studies conducted among veterinarians in the UK, the United States and Ireland.^2^, ^14^, ^15^, ^16^, ^17^, ^18^ Specifically, opportunities for improved provision of behavioural advice were notable. For instance, limited discussion during first puppy visits on how socialisation can be achieved may have long‐term repercussions on canine welfare, since appropriate socialisation, entailing gradual, pleasant exposure of a puppy to new experiences during the sensitive and juvenile period, is a major determinant of a dog's future behaviour.^43^, ^44^

Paucity in behavioural advice provision also emerged in discussions relating to dog–human aggression cases. Reference to offering first‐aid behavioural advice, such as guidelines on how to minimise the recurrence of incidents,^45^ was lacking. Instead, participants reported encouraging clients to seek behaviourist assistance, despite their general lack of confidence in available behaviourist options. This approach may negatively impact One Welfare, due to the delay in receiving crucial first‐aid advice and the financial cost deterring caregivers from engaging a behaviourist.^46^ Additionally, immediate advice to seek behaviourist support within an unregulated industry may result in clients receiving highly variable behavioural advice.

Where behavioural advice was provided, it appeared to follow minimal enquiry. This finding is consistent with previous research,^13^, ^14^, ^15^ and, together with deficiencies in behavioural advice provision, may delay appropriate case management. Findings from this study also suggest that dominance theory continues to inform some veterinary advice on canine behaviour. Dominance theory extrapolates findings about wolf social structure and communication to the relationship of the domestic dog with other dogs and people,^47^ and its application thus reflects the belief that mimicking wolf social structure by creating a clear dominance hierarchy between human caregiver and dog can resolve canine behaviour issues such as aggression.^47^ Although the relevance of dominance theory to domestic dogs has been hotly debated for many years, its use is nowadays largely considered inapplicable by behavioural experts.^48^, ^49^, ^50^, ^51^ Applying dominance theory can justify the use of positive punishment as a way of creating a dominance hierarchy,^48^, ^52^, ^53^ negatively affecting caregiver–dog interactions^54^ and potentially endangering dogs and people.^1^

Interestingly, advice based on dominance theory was only mentioned by male participants. This could relate to sex differences in empathy towards animals and attitudes towards VBM, which have been previously reported in the literature. Specifically, studies suggest that female veterinarians may exhibit greater empathy towards animals^55^, ^56^ and more favourable attitudes towards VBM compared to their male counterparts.^13^ While the generalisability of the findings reported in the literature is also hindered by various methodological limitations,^13^, ^55^, ^56^ the present study offers valuable insights in this context that may be worth exploring further.

In relation to the approach to the physical examination, foregoing it in situations where restraint was challenging could result in misdiagnosis and underdiagnosis.^57^ However, it may arguably reflect participants’ positive regard towards canine mental health and the decision to avoid excessive restraint and canine stress.^58^ Interestingly, long‐term behaviour management plans involving behaviour modification training for non‐cooperative dogs were generally not mentioned. Recommending such training could facilitate future veterinary examinations^59^ and improve veterinary experiences for dog, caregiver and veterinarian.^57^

Notwithstanding the above potential shortcomings, it is understandable that navigating canine behaviour issues can be challenging for general practice veterinarians, particularly in the absence of formal veterinary behavioural training and reliable external behavioural expertise to seek support from.

### Barriers to the practice of VBM

#### Knowledge gaps

In line with extant literature,^13^, ^14^, ^16^, ^17^, ^18^ participants' knowledge limitations regarding VBM were evident and, in cases, self‐declared. A degree of ambiguity in relation to VBM‐related terminology also emerged. For instance, the interchangeable use of the terms ‘trainer’ and ‘behaviourist’ by many respondents (defined in Table 4) may reflect a lack of clarity about these roles that could potentially result in client referral to an inappropriate service.^60^

**TABLE 4:** Distinguishing between the ‘trainer’ and the ‘behaviourist’.

Term/role	Definition
Trainer	A person who works solely with the dog and is that dog's handler. or A person who trains dogs and their handlers in order to prevent or manage problem behaviour.
Behaviourist	A person who works with the owner/handler to discover the aetiology of the problem behaviour and devise and implement a behaviour modification programme that is specific to that case.

*Source*: Information adapted from McBride.^60^

Knowledge limitations are likely to cause discomfort when addressing behavioural issues,^18^ as declared by eight out of 11 participants in this study, potentially contributing to reluctance to discuss behavioural issues.^2^ Furthermore, since knowledge is a potential determinant of attitude,^61^ limitations in knowledge of VBM and its relevance to veterinary practice may also have contributed to participants' views of VBM as external to veterinary medicine. Such views may further demotivate study and practice in the field.

The potential for VBM to be integrated into veterinary practice did not emerge, with participants referring to VBM mostly in terms of behavioural problem management rather than prevention. This may explain participants’ views of VBM as being time consuming. The concurrent existence of such views and feelings of time pressure in day‐to‐day veterinary practice may amplify the barrier to engaging in VBM. This underlines the importance of ensuring veterinary awareness of time‐effective ways of practising VBM within general practice. This may include veterinary provision of preventive behavioural advice and delegation of aspects of behavioural service provision to veterinary nurses.^45^, ^62^ However, effectively integrating aspects of VBM in general practice requires targeted education. While the compulsory inclusion of VBM in the clinical years of veterinary undergraduate curricula would be a positive step,^2^, ^17^, ^18^ it does not address the needs of those already in practice. Initiatives aimed at ensuring easy access to continuous professional development about VBM and its integration in general practice may be useful. Furthermore, access to interprofessional education could prepare undergraduate and qualified veterinarians for potential collaboration with allied veterinary professionals, including BSPs. Research into how such educational frameworks could be developed and implemented would be valuable in shaping a more integrated approach to behaviour cases.

#### Veterinarian–client communication

Opportunities for improved client communication emerged among some participants, in line with previous veterinarian‒client communication research.^63^, ^64^ These included the need for improvement in skills relating to expressing empathy and in framing potentially sensitive advice in a tactful way, such that behavioural advice could be provided to clients while minimising the risk, or the fear, of this being misinterpreted as judgement. Since adequate veterinarian‒client communication is regarded as vital for both veterinary and VBM practice,^18^, ^65^ the reported limitations in veterinarian–client communication represent a potential barrier to veterinary behavioural service provision.

#### Behavioural referral service limitations

Many participants expressed doubts about directing clients to non‐veterinary BSPs, citing concerns about the competence of BSPs, the prescription of medications by some non‐veterinary BSPs and mixed feedback from clients. These concerns highlight the difficulties veterinarians face in making informed decisions when external behaviour support is lacking. During the interview phase of this study, no Malta‐registered veterinarians were offering behaviour referral services, and no regulating body of non‐veterinary BSPs, to support interdisciplinary collaboration, exists in Malta to date. It was evident in participant narratives that this situation makes assisting patients with behavioural issues very challenging, and this contributed to a sense of helplessness and frustration. Moreover, since the Maltese Guide to Veterinary Professional Conduct does not discuss potential collaboration with allied veterinary professionals such as BSPs,^66^ it is understandable that reluctance to direct clients to BSPs may be further compounded. Furthermore, the generally limited rapport between veterinarians and existing BSPs may further contribute to mistrust,^67^ exacerbating the reluctance to engage in collaboration.

The non‐regulated status of BSPs in Malta also makes it difficult for veterinarians to determine BSP competence. Although this lack of regulation of BSPs is shared by various other countries, the importance of setting up regulatory frameworks for non‐veterinary animal behaviour professionals is being increasingly recognised, with organisations such as the International Association of Animal Behavior Consultants providing the opportunity for formal accreditation.^68^ The European College of Animal Welfare and Behavioural Medicine also provides a route for veterinarians specialising in behavioural medicine to be formally recognised as diplomates; however, there are relatively few behaviour medicine diplomates to date, and none practise in Malta.^69^ In the UK, governing bodies have been established to ensure minimum standards of competence for behaviour professionals, and the Royal Veterinary College of Veterinary Surgeons (RCVS) is working towards formal recognition of paraprofessionals in this area.^70^

Ultimately, having a national regulatory body that provides the public and veterinarians with a clear way of judging the competence of both veterinary and non‐veterinary BSPs would be desirable.^71^ Its absence can result in veterinarians feeling personally responsible for the choice of BSP, and the fear of getting this choice wrong was reflected in the reluctance of some participants to direct clients to a named BSP, or to direct them at all. Additional implications of this situation potentially include non‐referral of dogs requiring behavioural services, inadequate delegation procedures, for example, omission of medical history discussion, and directing clients to incompetent BSPs.

Where BSP regulation is pending, veterinarians could try to explore the credentials of available BSPs and gain awareness regarding important aspects that could better guide their choice of BSP.^72^ Additionally, reciprocal communication between veterinarians and BSPs upon initial contact and throughout the behaviour assessment process would be crucial in optimising One Welfare.^65^, ^73^

#### Participants’ beliefs

Participants' belief that experienced dog owners require less information during first puppy visits could lead to missed opportunities in providing guidance to clients. Literature suggests that it is owner knowledge of dog socialisation, rather than experience, that can reduce the development of behavioural problems.^74^, ^75^ Thus, it should be assumed that advice is useful to all owners, regardless of their experience.^75^

Another emergent belief was the futility of addressing aggressive behaviour in dogs who have displayed it since puppyhood. This belief may delay adequate behaviour management, resulting in progression of the problem and a poorer prognosis.^76^

### Study limitations

The findings of this study should be interpreted with caution. Prospective participants were aware of the study topic, making recruitment bias possible. The generalisability of the findings is also limited by the interview context, although it is argued that qualitative research should be concerned with ‘fittingness’ of data, rather than generalisability.^77^ Fittingness is the degree to which qualitative study findings ‘fit contexts outside the current research study situation’.^77^ One way of assessing the fittingness or applicability of this study's findings would be for future discussions to be held between the researchers and a number of veterinarians practising in Malta to gauge whether they consider the findings ‘meaningful and applicable in terms of their own experiences’.^77^ Moreover, while purposive sampling aimed to introduce variation, the demographic characteristics of respondents imposed certain limitations. Further quantitative studies would be valuable in assessing whether these findings are representative of veterinarians in Malta. Also, since one of the interviews was conducted in the Maltese language, language discrepancies may have occurred in the translation process.^78^ However, since the primary researcher is a native speaker of both languages, the researcher could refer back to the original Maltese language transcript during data interpretation, thus limiting miscommunication or loss of meaning. The presence of unconscious researcher biases cannot be ruled out, although a reflexive approach was employed to mitigate any potential impact of these biases.^40^ Interpretation of qualitative data is also subject to researcher influence,^79^ and thus, having a third party evaluate the coding in relation to the transcripts could have minimised researcher bias.^80^ Nevertheless, the use of a clear data analysis framework (template analysis) helped mitigate researcher influence through the systematic and structured analysis of the data.

Finally, the choice and use of certain terms in the interview could have influenced participant narratives. For instance, the term ‘specialist’ was used to refer to any person who offers animal behaviour services. However, veterinary regulatory bodies, such as the RCVS in the UK, lay out clear criteria to be achieved to be recognised as a specialist in any area of veterinary medicine.^81^ Similarly, the term ‘referral’ was used to refer to the practice of a veterinarian directing clients to any person who offers animal behaviour services. However, the RCVS uses the term ‘referral’ strictly in the context of a veterinarian referring a case to another veterinarian.^82^ Notwithstanding, participants did not appear confused or ask for clarification on the use of these terms during the interviews. The fact that the interviewer shares the same profession and cultural context as the participants offered a degree of insight as to what terms are generally used among general practice veterinarians in Malta in such contexts and what terms were thus likely to limit this potential misinterpretation of meaning.

## CONCLUSION

This study was the first to explore veterinary attitudes towards and practice of VBM using a qualitative interview approach, and the first exploring this field in Malta. There are indications that there may be opportunities for improvement in the practice of VBM among this study population. Several potential barriers to the practice of VBM emerged, including knowledge gaps among veterinarians, time constraints related to general veterinary practice, veterinarian–client communication barriers, limited availability of appropriate and trusted BSPs and a lack of regulation of BSPs.

This study highlights avenues where veterinarians may be able to institute changes in their practice that could positively impact One Welfare. These include changes in consultation content, such as the inclusion of provision of adequate preventive behavioural advice in first puppy visits,^75^ as well as potential changes in practice management, such as those aimed at relieving time pressure to better balance One Welfare with economic and logistical aspects.^83^

These findings also have relevance for policy makers. While the establishment of a regulatory body for BSPs that is recognised by government is a potentially complex undertaking, it would assist in improving behavioural service provision and satisfying One Welfare needs.^60^ Finally, since canine behavioural problems can lead to relinquishment,^18^ ensuring baseline practice of VBM by veterinarians and competency and regulation of BSPs, alongside a standardised protocol for interprofessional collaboration, could potentially assist in curbing shelter overpopulation.

## AUTHOR CONTRIBUTIONS

All contact with participants was made by Maria Debono. Maria Debono also drafted this research paper. Belinda Vigors and Amy Miele assisted Maria Debono throughout the process by providing guidance during study design, interview preparation, data analysis and reporting of findings. All the authors revised and approved the final version of this paper.

## CONFLICT OF INTEREST STATEMENT

Maria Debono started to offer a veterinary behaviour referral service in Malta as of April 2024, 2 years after the study was completed and the findings submitted as a dissertation as part of an MSc in Clinical Animal Behaviour.

## ETHICS STATEMENT

The project received ethical approval from the University of Edinburgh Royal (Dick) School of Veterinary Studies Human Ethical Review Committee (HERC) in November 2021 (HERC_766_21).

## Supporting information



Supporting Information

## Data Availability

Data available on request due to privacy/ethical restrictions.
