# Supplementary material for: Qualitative interview study exploring Maltese veterinarians’ practice of behavioural medicine
Source: Vet Rec. 2025 Jun 6;197(1):e5497. doi: 10.1002/vetr.5497 (PMC12232595; doi:10.1002/vetr.5497)
Supplement: Supplementary file 1 — Supporting Information [file VETR-197-e5497-s001.docx]

**Supplementary material**

Appendix 1: Framework for the development of a qualitative semi-structured interview guide, adapted from Kallio and colleagues (2016)^37^

| **Framework steps** | **Description** |
| --- | --- |
| 1. Identifying the prerequisites for using semi-structured interviews. | - - - - This study was concerned with how vets think and act in their daily lives and the reasons behind this. - Thus, a semi-structured interview approach, modelled on normal conversation, was deemed to be appropriate and was expected to increase the likelihood for the required information to be gained, as opposed to a formal question-answer approach. |
| 1. Retrieving and using previous knowledge. | - An extensive literature review was carried out to gain understanding of the topic under study. - This was complemented with personal knowledge of the subject gained through working as a veterinary surgeon in the same country and cultural context as participants. |
| 1. Formulating the preliminary semi-structured interview guide. | - Knowledge gained in step 2 served as a basis for the identification of key themes to be included in the interview guide. These can be viewed as part of the interview guide in Appendix 2. - A number of open-ended questions and probes for each key theme were formulated. |
| 1. Pilot testing the interview guide. | - Guide was pilot tested with 2 non-vets for clarity. Amendments in wording and choice of questions followed. - The guide was then pilot tested with 2 non-participating small animal vets practising outside of Malta. - These pilot interviews were transcribed and analysed, and amendments were made accordingly. |
| 1. Presenting the complete semi-structured interview guide. | - The interview guide used can be found in supplementary material (Appendix 2). |

Appendix 2: Interview Guide

**Introduction**

-**Thank you** for choosing to participate in this study.

-Brief **overview of study aims**

-Explain **interview “structure**”-normal conversation, no right/wrong answer, right of stopping interview/refusing to answer

-Inform participant when recording starts.

**Interview guide: Key themes and main interview questions.**

**(Order in which questions are posed may vary, depending on interviewee’s responses)**

| **Key Themes** | **Vets’ attitudes towards canine mental health and field of veterinary behaviour medicine** | **Awareness and knowledge of role in prevention/early intervention**  **Practise of behavioural medicine**  **Relative interest in behaviour field** | **Barriers to behavioural service provision** |
| --- | --- | --- | --- |
| **Interview questions** | Can you tell me what the term ‘canine wellbeing’ means to you?  Probe: Can you please describe some of the things which you feel are essential to canine wellbeing.  (if canine mental health is mentioned, what factors do you think affect canine mental health?)  Could you tell me about a time when a dog would not stay still during a clinical exam?  Probe: If aggression is discussed: Could you also tell me about other similar situations which did not involve aggression.  Can you recall a situation where you feared for your own safety during a canine consult?  Probes: What did you do to be able to examine the dog?  Did you take any measures to ensure personnel and client safety in future consults with that dog?  Can you recall a time when you brought an owner and their dog into the treatment area?  Probe:  -for a routine procedure?  -What were your reasons for doing this?  Or- Can you tell me of other reasons why you may bring the owners into the treatment area?  If you never brought owners into treatment area, is there any particular reason? | What do you think, from your experience, are the main reasons for dogs being given up by their owners?  Probe: As a vet, do you believe there is anything you can do to reduce the likelihood of dogs being given up?  Can you describe your approach to a first puppy visit?  Probe: Can you tell me what general advice you would give to the owner during their first visit. | Can you recall a time when a dog owner visited for a behavioural issue?  Probe: Can you tell me more about it.  Could you tell me about a time when an owner visited because their dog had bitten a person?  Could you tell me about a time where you referred a case to a behaviour specialist?  Probe:  Do you have any concerns or reservations about referring behaviour cases to behaviour specialists in Malta?  Could you describe your comfort level when dealing with canine behaviour issues?  Probe: What do you think has contributed to how you feel about dealing with behaviour issues?  How does your comfort in dealing with behaviour issues compare to dealing with medical issues” – what are the reasons for this, do you think? |

**General probing statements:**

| Can you please clarify what you mean by the term “…..”? | What led you to do “that”? |
| --- | --- |
| Could you tell me more about “X” | Could you share some examples? |
| Why do you think that is the case? | How did you feel at the time? |
| Could you elaborate more or give reasons |  |

**Anticipated risk situations and appropriate ways of responding**

| **Risk** | **Mitigation** |
| --- | --- |
| Interviewee starts to disclose sensitive information | Steer conversation away from that subject: (“Could we please change subject now and talk more about X..”) |
| Interviewee appears uncomfortable when a certain question is posed | Ask participant “Would you prefer to move on to a different question?” |
| Interviewee appears distracted, uninterested or wanting to leave | Ask participant “Would you like to continue at another time?” |

**Conclusion:**

Thank you for your time.

Appendix 3: Final coding template

| Name | Files | References |
| --- | --- | --- |
| 1. **Differing views surrounding referral of behaviour cases** | 0 | 0 |
| Comfort when referring depends on issue and available trainer | 1 | 1 |
| Confusing terminology trainer or behaviourist obedience or behavioural issue | 1 | 2 |
| Clear use of terminology dog trainer vs behaviourist | 1 | 1 |
| Keen to refer | 1 | 2 |
| General reasons for referral | 0 | 0 |
| Visiting the place where the dog is kept is important | 1 | 2 |
| Reasons for referral to a particular behaviourist or dog trainer | 4 | 6 |
| Not comfortable referring | 2 | 2 |
| Dangerous practice | 2 | 5 |
| Limited knowledge of what the referral options are | 4 | 8 |
| Limited positive client feedback after going to trainer or behaviourist | 3 | 4 |
| No relationship with trainer or behaviourist | 1 | 2 |
| Questionable competence | 9 | 17 |
| Scant referral service providers | 4 | 7 |
| Trainers offer classes, not individual approach | 2 | 2 |
| 1. **Emotions surrounding the practice of veterinary behavioural medicine** | 0 | 0 |
| Distress and concern | 1 | 4 |
| Difficulty balancing physical health and mental health | 2 | 2 |
| Euthanasia is sometimes inevitable | 1 | 1 |
| Fear and doubt | 5 | 7 |
| Frustration, anger and sadness | 4 | 14 |
| Refusing euthanasia | 3 | 8 |
| Stuck and unsupported | 6 | 12 |
| Perceived self-efficacy | 2 | 3 |
| Running out of patience...and time | 1 | 1 |
| Taking getting bitten lightly | 5 | 9 |
| The owner is mostly to blame | 3 | 16 |
| 1. **Potential barriers to the practice of veterinary behavioural medicine** | 0 | 0 |
| Limited demand of service from vets | 3 | 9 |
| Limited rapport between client and vet | 0 | 0 |
| Limited empathy | 2 | 5 |
| Limited support from owner | 1 | 6 |
| Limited time | 6 | 20 |
| Behaviour issues take time | 1 | 2 |
| Belief in the need for a home visit | 2 | 2 |
| Limited time to gain knowledge | 1 | 1 |
| Making assumptions | 2 | 2 |
| Most dogs only visit once a year | 1 | 1 |
| Self-declared limitations | 2 | 5 |
| Limited knowledge | 5 | 7 |
| Behaviour not well covered in undergraduate degree | 7 | 11 |
| No space for veterinary behavioural medicine in the already overloaded veterinary curriculum | 2 | 2 |
| Experience without knowledge | 1 | 1 |
| More comfortable with other medical cases | 8 | 12 |
| Discomfort leading to lack of enquiry | 1 | 1 |
| Limited relative interest in the field | 3 | 3 |
| Not 100% comfortable dealing with behavioural issues | 4 | 8 |
| Not 100%comfortable but belief in being best person to give advice | 1 | 3 |
| Veterinary surgeons' opinions and perceptions | 0 | 0 |
| A dog is born with a character | 2 | 4 |
| Behavioural issues as a slight inconvenience | 1 | 1 |
| Belief in dominance and confusion about learning theory | 6 | 27 |
| Previous dog ownership is a measure of owner's competence | 4 | 4 |
| Seeing behaviour practice as a completely separate speciality with limited potential for integration | 2 | 3 |
| Small dogs are low risk | 1 | 1 |
| Too late to intervene | 2 | 2 |
| Veterinary perceptions of dog owners | 2 | 2 |
| Limited owner knowledge and education | 4 | 5 |
| Low owner motivation to address behavioural issues | 7 | 9 |
| Owners not acknowledging problem behaviour | 2 | 2 |
| Owners may find advice offensive | 5 | 8 |
| People do not listen to vets | 3 | 5 |
| 1. **Practice of veterinary behavioural medicine** | 1 | 2 |
| Aggressive behaviour | 0 | 0 |
| Believed potential causes of aggressive behaviour | 0 | 0 |
| Dominance and hierarchy theories | 1 | 1 |
| Fear aggression | 2 | 3 |
| Guarding | 1 | 1 |
| Lack of exercise and owner dog interaction | 1 | 1 |
| Pain can cause biting in consult room | 2 | 2 |
| Reaching a solution | 0 | 0 |
| Advising about aggressive behaviour | 0 | 0 |
| Behaviour modification alongside herbal remedies or drugs | 2 | 2 |
| Behaviour modification tips | 2 | 5 |
| Expressing concern, but no real advice | 3 | 5 |
| Limited support and mirky advice | 3 | 11 |
| People-focused advice | 0 | 0 |
| Dog training collar No physical damage and it works, at times | 1 | 2 |
| Rehoming or euthanasia | 4 | 8 |
| Safety | 1 | 2 |
| Doubt about the use of behaviour modifying drugs and neutraceuticals | 2 | 4 |
| Referral | 3 | 4 |
| Through neutering | 2 | 2 |
| Approach to canine physical exam | 0 | 0 |
| Covering eyes helps | 1 | 1 |
| Factors affecting approach to physical exam | 0 | 0 |
| Being practical | 5 | 13 |
| Dog and handler safety | 5 | 9 |
| Dog factors - condition, size, behaviour | 8 | 24 |
| Pain | 2 | 3 |
| Ease of future visits | 2 | 4 |
| Limited assistance during consult | 1 | 2 |
| Owner factors | 7 | 12 |
| Distancing or removing the owner can help | 3 | 5 |
| Muzzles | 11 | 27 |
| Physical restraint | 8 | 20 |
| Owner restraint leads to biting | 1 | 1 |
| Pre-consult advice to facilitate exam | 2 | 2 |
| Sedation | 7 | 18 |
| Slow and gentle | 4 | 7 |
| Time spent building positive associations is important | 1 | 4 |
| Talking to the dog helps | 1 | 1 |
| Using high pitched voice | 1 | 1 |
| Behavioural cases are uncommon vs behavioural cases present frequently | 10 | 15 |
| Canine mental health and the veterinary visit | 2 | 6 |
| Defining canine wellbeing | 9 | 17 |
| Factors affecting canine well being | 0 | 0 |
| Antropomorphism as a threat to canine physical and mental wellbeing | 1 | 3 |
| Mental health | 0 | 0 |
| Dogs are social creatures | 5 | 7 |
| human-dog interaction | 5 | 13 |
| effect of owner | 1 | 1 |
| Mourning | 1 | 1 |
| Socialisation as a puppy | 2 | 2 |
| Exercise and environmental enrichment for mental health | 5 | 6 |
| Genetics, personality and mental health | 5 | 6 |
| Having a routine | 1 | 1 |
| Previous experiences | 2 | 3 |
| Physical health | 1 | 2 |
| Physical health needs | 3 | 6 |
| Mental health is important | 6 | 9 |
| Measures taken to reduce canine stress during visit | 2 | 4 |
| Asking owner to stay or leave for euthanasia | 1 | 2 |
| Calling the nurse | 1 | 1 |
| Calming plug ins | 1 | 1 |
| Changing location and attire | 1 | 1 |
| Clinic management practices | 2 | 4 |
| Examining on the ground, not on a table | 2 | 2 |
| Give dog time to settle, speak to owner first | 3 | 3 |
| Giving treats and cuddles | 3 | 7 |
| Music | 1 | 1 |
| Not a full physical exam | 5 | 5 |
| Noting gender preference | 1 | 2 |
| Same pet, same vet | 2 | 2 |
| Sedation as a stress reducing measure | 2 | 3 |
| The owner's presence for the dog in the clinic | 9 | 26 |
| only one mentioned taking dog away from owner for practical reasons | 1 | 1 |
| Vets and vet clinics are stressful for dogs | 7 | 12 |
| Covid effects | 3 | 7 |
| Dog breed or size can affect practice of veterinary behavioural medicine | 9 | 25 |
| Enquiring about behaviour or behaviour issues | 7 | 12 |
| Following up behavioural issues | 2 | 4 |
| First puppy visit | 2 | 6 |
| Behaviour related advice | 10 | 54 |
| General approach | 5 | 11 |
| General preventive advice | 5 | 12 |
| General approach to use of behaviour modifying drugs | 5 | 10 |
| Labels | 3 | 3 |
| Limited behavioural advice for breeders | 1 | 2 |
| Neutering | 11 | 54 |
| Pre-adoption advice | 2 | 6 |
| Preparing owner for procedure is important | 1 | 1 |
| Referral procedure | 1 | 1 |
| Behaviourist referral as a knee jerk reaction | 6 | 7 |
| Clients do the research | 4 | 7 |
| Limited communication between referring vet and trainer and or behaviourist | 5 | 10 |
| One vet mentioned discussing with trainer | 1 | 1 |
| Realisation of importance of communication with behaviourist or trainer | 1 | 1 |
| Some vets try to manage first then refer | 2 | 2 |
| Vet guides to specific trainer, behaviourist | 1 | 2 |
| Separation related behaviour | 3 | 9 |
| 1. **Various reasons for canine relinquishment** | 11 | 37 |
| Lack of enforcement | 5 | 10 |
| Limited tolerance for owner reasons for relinquishment | 2 | 9 |
| 1. **Veterinary surgeons' view of their role** | 0 | 0 |
| As animal welfare advocates in society at large | 2 | 2 |
| As educators of clients | 6 | 8 |
| Awareness of the importance of educating | 4 | 10 |
| Educating about legal issues | 1 | 1 |
| Limited awareness of importance of offering behavioural preventive advice | 1 | 2 |
| As providers of a behavioural service | 0 | 0 |
| Dealing with behavioural issues is not my job. | 2 | 4 |
| I am not a behaviourist | 7 | 15 |
| Too much responsibility | 1 | 4 |
| We give valuable advice | 2 | 2 |
